# Molecular predictors of post-transplant survival in acute myeloid leukemia

**DOI:** 10.1038/s41408-017-0027-6

**Published:** 2017-12-13

**Authors:** Tong Qin, Sun Wu, Hongmian Zhao, Keman Xu, Huaping Fu, Zhiheng Cheng, Yifan Pang, Yu Han, Li Chen, Chao Wang, Yijie Zhang, Xiaoyan Ke, Kailin Xu, Jinlong Shi, Lin Fu

**Affiliations:** 1 Department of Hematology, Huaihe Hospital of Henan University, Kaifeng, 475000 China; 2 Department of Hematology and Lymphoma Research Center, Peking University, Third Hospital, Beijing, 100191 China; 3 Department of Hematology, The First Affiliated Hospital of Xinxiang Medical University, Weihui, 453100 China; 4 Northeastern University, Boston, MA 02115 USA; 5 Department of Nuclear Medicine, Chinese PLA General Hospital, Beijing, 100853 China; 6 Translational Medicine Center, Huaihe Hospital of Henan University, Kaifeng, 475000 China; 7 Department of Medicine, Wil-liam Beaumont Hospital, Royal Oak, MI 48073 USA; 8 Department of Respiratory, Huaihe Hospital of Henan University, Kaifeng, 475000 China; 9 Department of Hematology, The Affiliated Hospital of Xuzhou Medical University, Xuzhou, 221002 China; 10 Department of Biomedical Engineering, Chinese PLA General Hospital, Beijing, 100853 China; 11 Department of Medical Big Data, Chinese PLA General Hospital, Beijing, 100853 China

Acute myeloid leukemia (AML) is a heterogeneous disease. Based on risk stratification at diagnosis, patients with AML either receive consolidation chemotherapy or undergo allogeneic hematopoietic stem cell transplantation (allo-HSCT) after attaining initial remission. It has been shown that cytogenetic abnormalities at diagnosis are associated with outcome after post remission therapy, including allo-HSCT^1^. Based on cytogenetic risk stratification, Koreth et al.^2^ undertook a systematic review and meta-analysis of prospective trials, and concluded that allo-HSCT had significant relapse-free survival and overall survival (OS) benefit for intermediate- and for poor-risk AML, but not for good-risk AML in first complete remission. A growing number of recurrent genetic abnormalities have been recognized in the revised 2016 World Health Organization (WHO) classification of AML^3^. Acquired recurrent genetic abnormalities at diagnosis are among the most important independent factors used for diagnosis and prognostic stratification, and deepens our understanding of the disease pathogenesis^4^. Whether recurrent genetic abnormalities are also important markers that imply response to allo-HSCT is unknown. To address this question, we examined samples from patients with AML who had undergone allo-HSCT to determine whether recurrent genetic abnormalities were associated with long-term outcome after transplantation.

This is a retrospective study. A total of 78 patients who received allo-HSCT for AML (derived from The Cancer Genome Atlas (TCGA) database (https://cancergenome.nih.gov/) were included in our study. Median age of the patients was 51 years (range, 18–72 years) and 45 (58%) patients were males. FAB subtype data were available for 77 patients, which included M0 (*n* = 10; 13%), M1 (*n* = 23; 30%), M2 (*n* = 20; 26%), M3 (*n* = 3; 4%), M4 (*n* = 14; 18%), M5 (*n* = 5; 6%), M6 (*n* = 1; 1%), and M7 (*n* = 1; 1%). The median WBC count at diagnosis for the entire cohort was 28.55 × 10^9^/L (range, 0.6 × 10^9^/L–223.8 × 10^9^/L), and five patients (6%) had WBC count of ≥100 × 10^9^/L at diagnosis. The median bone marrow blast at diagnosis for the entire cohort was 71.5% (range, 30–100%), and 65 patients (83%) had a bone marrow blast percentage of ≥50%. Cytogenetic data were available for 77 patients. Eight patients (10%) belonged to the good-risk, 47 (60%) to the intermediate-risk, and 22 (28%) to the poor-risk groups. Donors included human leukocyte antigen (HLA)-identical matched related donors (MRD, *n* = 33), HLA-identical matched unrelated donors (MUD, *n* = 43), and haploidentical related donors (HRD, *n* = 2). Twenty-seven patients did not achieve complete remission (CR) before transplantation. The median numbers of recurrent genetic mutations at diagnosis was 5 (range, 0–12). Seventy-seven patients had mutations in one or more genes. *NPM1* was the most frequently mutated gene (*n* = 21, 27%), followed by *DNMT3A* (*n* = 19, 24%), *FLT3-ITD* (*n* = 17, 22%), *IDH1* (*n* = 11, 14%), *IDH2* (*n* = 9, 12%), *RUNX1* (*n* = 9, 12%), *WT1* (*n* = 9, 12%), *CEBPA* (*n* = 8, 10%), *MYH11-CBFB* (*n* = 5, 6%), *MLL-translocation* (*n* = 5, 6%), *MLL-PTD* (*n* = 4, 5%), *TET2* (*n* = 4, 5%), *TP53* (*n* = 4, 5%), *KIT* (*n* = 4, 5%), *U2AF1* (*n* = 3, 4%), *STAG2* (*n* = 3, 4%), *ASXL1* (*n* = 2, 3%), *EZH2* (*n* = 2, 3%), *BCR-ABL1* (*n* = 2, 3%), and *NUP98-NSD1* (*n* = 2, 3%). Clinical and molecular characteristics are summarized in Supplementary Table 1.

To assess the prognostic significance of these mutations, we focused on 14 genetic mutations which were detected in 5% or more of the patient population (*NPM1*, *DNMT3A*, *FLT3-ITD*, *IDH1*, *IDH2*, *RUNX1*, *WT1*, *CEBPA*, *FLT3-ITD* ^*+*^ */NPM1* ^*-*^, *MYH11-CBFB*, *MLL-PTD*, *TET2*, *TP53*, and *KIT*). Factors including age (<60 vs. ≥60), WBC count (<100 × 10^9^/L vs. ≥100 × 10^9^/L), bone marrow blast percentage (<50% vs. ≥50%), cytogenetic risk (poor vs. others), donor type (MRD vs. MUD), disease state (CR vs. not in CR) as well as numbers of recurrent genetic mutations (<5 vs. ≥5). The results are summarized in Table 1. Based on univariate analyses, *MLL-PTD* was unfavorable for both OS (*P* = 0.024; Fig. 1g) and EFS (*P* < 0.001; Fig. 1h). Mutations in *RUNX1* (*P* = 0.029; Fig. 1c) and *TP53* (*P* = 0.011; Fig. 1d) negatively affected OS, while mutations in *WT1* (*P* = 0.033; Fig. 1e) were identified as unfavorable for EFS. Patients with genotype “*FLT3-ITD* ^*+*^ */NPM1* ^*-*^” showed a trend of poor EFS compared with those without the cytogenetic characteristic, but it did not reach statistical significance (*P* = 0.089; Fig. 1f). OS also appeared shorter in patients who did not achieve CR before transplantation (*P* = 0.051; Fig. 1a) and those having ≥5 recurrent genetic mutations (*P* = 0.071; Fig. 1b) whereas statistical significance was not achieved. Other clinical parameters, including age, WBC count, bone marrow blast percentage, cytogenetic risk, and donor type, were not associated with survival.

**Table 1:** Univariate and multivariate analysis for EFS and OS

	Univariate analysis		Multivariate analysis	
	*P*	Log rank *χ* ^2^ test	*P*	HR (95% CI)
OS				
Age (<60 vs. ≥60 years)	0.200	1.640	0.451	0.783 (0.414–1.481)
WBC (<100×10^9^/L vs. ≥100×10^9^/L)	0.429	0.626	0.211	0.495 (0.164–1.490)
BM blast (<50% vs. ≥50%)	0.710	0.138	0.790	0.908 (0.445–1.851)
Cytogenetic risk (poor vs. others)	0.737	0.113	0.949	0.978 (0.489–1.921)
Disease state (CR vs. not in CR)	0.051	3.792	0.096	0.619 (0.352–1.089)
Mutated recurrent genes (<5 vs. ≥5)	0.071	3.271	0.348	0.741 (0.396–1.387)
RUNX1	0.029	4.768	0.199	0.569 (0.241–1.345)
WT1	0.196	1.675	0.276	0.624 (0.267–1.457)
TP53	0.011	6.482	0.012	0.202 (0.059–0.700)
MLL-PTD	0.024	5.096	0.060	0.316 (0.095–1.050)
EFS				
Age (<60 vs. ≥60 years)	0.907	0.014	0.886	0.953 (0.497–1.828)
WBC (<100×10^9^/L vs. ≥100×10^9^/L)	0.571	0.321	0.331	0.584 (0.198–1.726)
BM blast (<50% vs. ≥50%)	0.711	0.137	0.631	1.191 (0.548–2.428)
Cytogenetic risk (poor vs. others)	0.901	0.015	0.756	1.115 (0.561–2.215)
Disease state (CR vs. not in CR)	0.311	1.026	0.545	0.842 (0.482–1.470)
Mutated recurrent genes (<5 vs. ≥5)	0.528	0.399	0.873	0.951 (0.517–1.753)
RUNX1	0.281	1.161	0.777	0.879 (0.359–2.152)
WT1	0.033	4.521	0.138	0.509 (0.208–1.242)
TP53	0.245	1.353	0.167	0.425 (0.126–1.430)
MLL-PTD	0.000	14.865	0.016	0.204 (0.056–0.746)

The variables selected in the Cox proportional hazard model: age, WBC count, bone marrow blast, cytogenetic risk, disease state, mutated recurrent genes, and mutations (including mutations with frequency ≥5% and have poor OS or EFS based on univariate analyses: RUNX1, WT1, TP53, and MLL-PTD)

*BM* bone marrow, *CR* complete remission, *EFS* event-free survival, *HR* hazard ratio, *OS* overall survival, *WBC* white blood cell

**Fig. 1:**
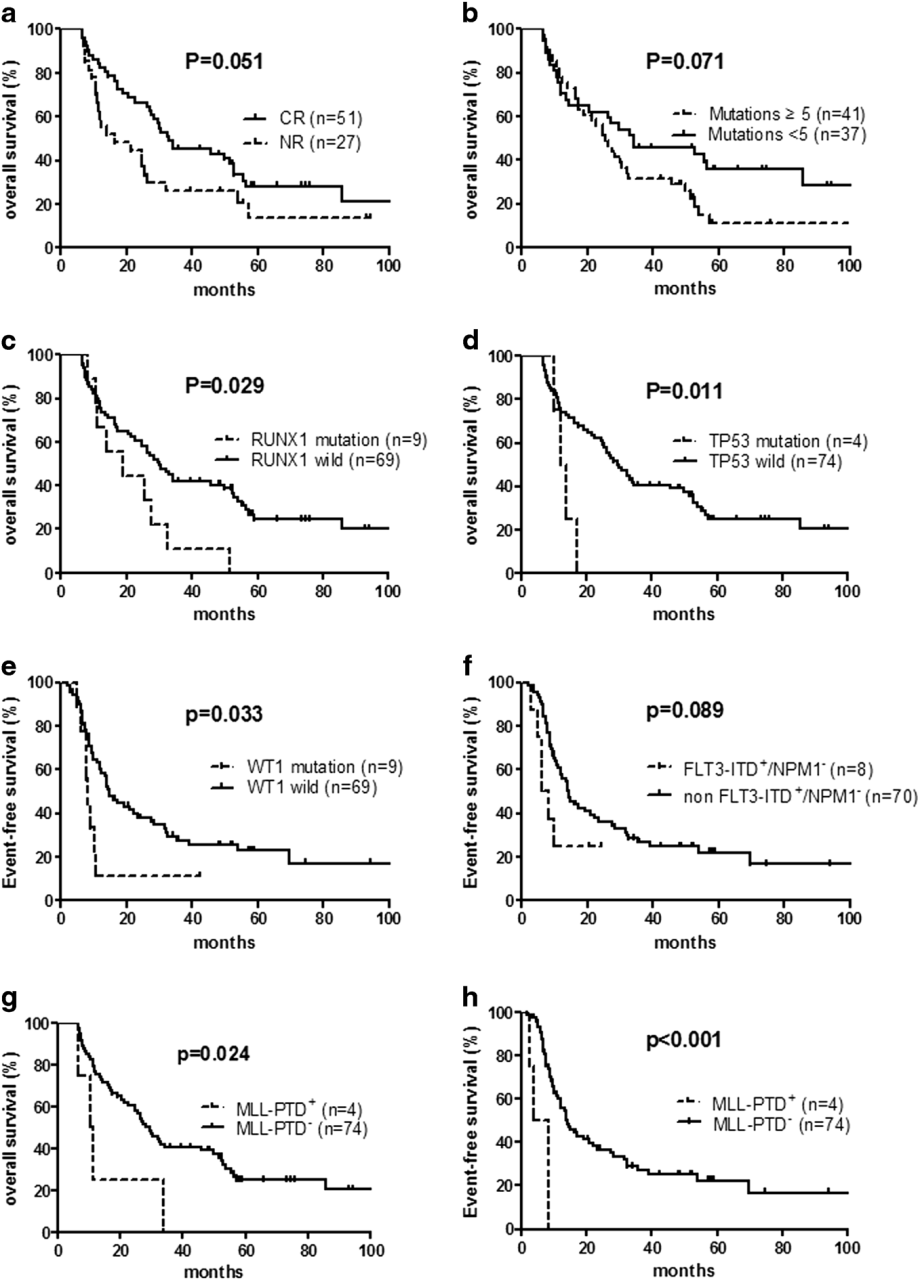
Kaplan–Meier curves of OS and EFS **a** Patients did not achieve CR before transplantation tended to have shorter OS than those transplanted in CR. **b** Patients with ≥5 mutations appeared to have shorter OS than those with <5 mutations. **c**, **d** Patients with RUNX1 and TP53 mutations had worse OS than wild-type groups. **e** Patients with WT1 mutations had worse EFS than wild-type groups. **f** Patients with genotype “mutated FLT3-ITD without NPM1” showed poor EFS compared with those without these mutations. **g**, **h** Patients with MLL-PTD mutations had worse OS and EFS than wild-type groups. Overall survival (OS) and event-free survival (EFS) were stratified by univariate prognostic factors. *P*-value was estimated by the log-rank test

We conducted multivariate COX regression analyses to identify independent risk factor for OS and/or EFS in the cohort. The four recurrent genetic mutations with demonstrated adverse effects on OS and/or EFS (*RUNX1*, *WT1*, *TP53*, and *MLL-PTD*, each of which showed significant associations with OS/EFS) and age (<60 vs. ≥60), WBC count (<100 × 10^9^/L vs. ≥100 × 10^9^/L), bone marrow blast percentage (<50% vs. ≥50%), cytogenetic risk (poor vs. others), disease state (CR vs. not in CR), as well as number of recurrent genetic mutations (<5 vs. ≥5) were incorporated in the analysis. Age (<60 vs. ≥60), WBC count (<100 × 10^9^/L vs. ≥100 × 10^9^/L), bone marrow blast (<50% vs. ≥50%), cytogenetic risk (poor vs. others), and number of recurrent genetic mutations (<5 vs. ≥5) had similar OS and EFS. *TP53* mutation was an independent risk factor for OS (HR, 0.202; 95% confidence interval, CI, 0.059–0.700, *P = *0.012). *MLL-PTD* was an independent risk factor for EFS (HR, 0.204; 95% CI, 0.056–0.746, *P = *0.016). The results of the multivariate analysis are summarized in Table 1.

In this study, we examined the prognostic significance of recurrent genetic mutations and other clinical parameters in post allo-HSCT AML patients. Previous studies had controversial findings about how *FLT3-ITD* mutations affected the prognosis of AML patients after allo-HSCT^5,6^. *RUNX1* mutations were found in 8 and 16% of younger and older patients with cytogenetically normal-AML (CN-AML), respectively, and they had lower CR rates and shorter disease-free survival (DFS), OS, and EFS than wild-type *RUNX1* patients^7^. It was found in a study that patients with WT1 mutations had shorter DFS and OS than patients with wild-type *WT1*(ref. 8). Compared with these studies, our results show that mutations in these genes were not associated with survival. We assumed that allogeneic hematopoietic stem cell transplantation in our cohort might have reversed the unfavorable influences of mutations in these genes.

A recent study observed no significant difference in either OS or DFS between *MLL-PTD* ^*+*^ and *MLL-PTD* ^*−*^ patients^9^. The authors postulated that intensive consolidation therapy, which included autologous stem cell transplantation in first complete remission, might have contributed to the better outcome of this historically poor-prognosis group of CN-AML patients. *TP53* gene mutations have been associated with monosomal karyotype and complex karyotype in myeloid malignancies^10^. The karyotype of the leukemic cells is by far the strongest prognostic factor for both response to induction therapy and survival^11,12^. A recent analysis of 858 AML patients demonstrated poor OS in patients with *TP53* mutation^13^. In our study, we demonstrated that the *TP53* and *MLL-PTD* mutations were independent predictors for inferior survival in post allo-HSCT patients. Our analyses indicated that evaluating the mutational status of the *TP53* and *MLL-PTD* genes would be necessary before planning allo-HSCT for AML patients.

Older age was traditionally associated with poorer outcomes in AML patients, but age has not been shown to be the most important predictor for either transplant-related mortality or resistance to therapy. Our study concurred with the previous study by showing that the OS and EFS of patients ≥60-year-old were similar to those <60-year-old and age was not an independent prognostic factor in multivariate analysis. Quality of life is an important outcome for hematopoietic cell transplantation recipients. Particularly physical functioning and functional well-being may provide independent prognostic information beyond standard clinical measures in allo-HSCT recipients^14^.

Previous studies suggest that heavier mutation burdens might be associated with poorer prognosis in myelodysplastic syndromes^15^. It is possible that mutation burden also influences the survival of AML patients. We found that OS appeared to be shorter in patients having mutated recurrent genes ≥5 based on univariate analyses, but did not show statistical significance. Further studies are warranted to validate this hypothesis.

Our study has two important limitations. First, the relatively small number of patients is the major limitation of our study. Retrospective study designs are generally considered inferior to prospective study designs; this is the second limitation of our retrospective analysis. Nevertheless, our data suggest that assessing *TP53* and *MLL-PTD* mutational status may be valuable for predicting survival in post allo-HSCT AML patients.

## Electronic supplementary material

Supplementary table 1

## References

[1] Kim DH (2005). Parameters for predicting allogeneic PBSCT outcome of acute myeloid leukemia: cytogenetics at presentation versus disease status at transplantation. Ann. Hematol..

[2] Koreth J (2009). Allogeneic stem cell transplantation for acute myeloid leukemia in first complete remission: systematic review and meta-analysis of prospective clinical trials. JAMA.

[3] Arber DA (2016). The 2016 revision to the World Health Organization classification of myeloid neoplasms and acute leukemia. Blood.

[4] Papaemmanuil E (2016). Genomic classification and prognosis in acute myeloid leukemia. N. Engl. J. Med..

[5] Savani BN (2010). Transplantation in AML CR1. Blood.

[6] Sengsayadeth S (2012). Allo-SCT for high-risk AML-CR1 in the molecular era: impact of FLT3|[sol]|ITD outweighs the conventional markers. Bone Marrow Transplant..

[7] Mendler JH (2012). RUNX1 mutations are associated with poor outcome in younger and older patients with cytogenetically normal acute myeloid leukemia and with distinct gene and MicroRNA expression signatures. J. Clin. Oncol..

[8] Paschka P (2008). Wilms’ tumor 1 gene mutations independently predict poor outcome in adults with cytogenetically normal acute myeloid leukemia: a Cancer and Leukemia Group B Study. J. Clin. Oncol..

[9] Whitman SP (2007). Long-term disease-free survivors with cytogenetically normal acute myeloid leukemia and MLL partial tandem duplication: a Cancer and Leukemia Group B Study. Blood.

[10] Breems DA (2008). Monosomal karyotype in acute myeloid leukemia: a better indicator of poor prognosis than a complex karyotype. J. Clin. Oncol..

[11] Grimwade D (2001). The clinical significance of cytogenetic abnormalities in acute myeloid leukaemia. Best Pract. Res. Clin. Haematol..

[12] Mrozek K, Heerema NA, Bloomfield CD (2004). Cytogenetics in acute leukemia. Blood Rev..

[13] Stengel A (2016). The impact of TP53 mutations and TP53 deletions on survival varies between AML, ALL, MDS and CLL: an analysis of 3307 cases. Leukemia.

[14] Hamilton BK (2015). Prognostic significance of pre-transplant quality of life in allogeneic hematopoietic cell transplantation recipients. Bone Marrow Transplant..

[15] Papaemmanuil E (2013). Clinical and biological implications of driver mutations in myelodysplastic syndromes. Blood.

