# Supplementary material for: Molecular predictors of post-transplant survival in acute myeloid leukemia
Source: Blood Cancer J. 2017 Dec 13;7(12):641. doi: 10.1038/s41408-017-0027-6 (PMC5802651; doi:10.1038/s41408-017-0027-6)
Supplement: Supplementary file 1 — Supplementary table 1 [file 41408_2017_27_MOESM1_ESM.docx]

Supplementary table1: Comparisons of clinical and molecular characteristics for the 78 AML patients

| Variable | Median (range) or N (%) |
| --- | --- |
| No. of patients | 78 |
| Age, years | 51 (18–72) |
| <60 years | 57(73) |
| ≥60 years | 21(27) |
| Gender |  |
| Female | 33(42) |
| Male | 45(58) |
| Median WBC count at diagnosis, ×10^9^/L | 28.55 (0.6–223.8) |
| <100×10^9^/L | 73(94) |
| ≥100×10^9^/L | 5(6) |
| BM blast at diagnosis, % | 71.50(30-100) |
| <50% | 13(17) |
| ≥50% | 65(83) |
| AML FAB subtype |  |
| M0 | 10(13) |
| M1 | 23(30) |
| M2 | 20(26) |
| M3 | 3(4) |
| M4 | 14(18) |
| M5 | 5(6) |
| M6 | 1(1) |
| M7 | 1(1) |
| Cytogenetic risk at diagnosis |  |
| Good prognosis | 8(10) |
| Intermediate prognosis | 47(60) |
| Poor prognosis | 22(28) |
| Unknown | 1(1) |
| Donor type |  |
| MRD | 33 (42) |
| MUD | 43 (55) |
| Haploidentical donor | 2 (3) |
| Disease state before transplantation |  |
| CR | 51(65) |
| not in CR | 27(35) |
| Mutated recurrent genes | 5(0-12) |
| <5 | 37(47) |
| ≥5 | 41(53) |

Supplementary table1, continue

| Variable | Median(range) or N (%) |
| --- | --- |
| RUNX1 |  |
| Mutated | 9(12) |
| Wild-type | 69(88) |
| WT1 |  |
| Mutated | 9(12) |
| Wild-type | 69(88) |
| KIT |  |
| Mutated | 4(5) |
| Wild-type | 74(95) |
| TET2 |  |
| Mutated | 4(5) |
| Wild-type | 74(95) |
| TP53 |  |
| Mutated | 4(5) |
| Wild-type | 74(95) |
| U2AF1 |  |
| Mutated | 3(4) |
| Wild-type | 75(96) |
| STAG2 |  |
| Mutated | 3(4) |
| Wild-type | 75(96) |
| ASXL1 |  |
| Mutated | 2(3) |
| Wild-type | 76(97) |
| EZH2 |  |
| Mutated | 2(3) |
| Wild-type | 76(97) |
| DNMT3A |  |
| Mutated | 19(24) |
| R882 | 10 |
| Non-R882 | 9 |
| Wild-type | 59(76) |
| IDH1 |  |
| R132 | 11(14) |
| Wild-type | 67(86) |
| IDH2 |  |
| Mutated | 9(12) |
| R140 | 7 |
| R172 | 2 |
| Wild-type | 69(88) |

Supplementary table1, continue

| Variable | Median(range) or N (%) |
| --- | --- |
| CEBPA |  |
| Mutated | 8(10) |
| Single-mutated | 5(6) |
| Double-mutated | 3(4) |
| Wild-type | 70(90) |
| FLT3-ITD/NPM1 status |  |
| FLT3-ITD^+^/NPM1^+^ | 9(12) |
| FLT3-ITD^+^/NPM1^-^ | 8(10) |
| FLT3-ITD^-^/NPM1^+^ | 12(15) |
| FLT3-ITD^-^/NPM1^-^ | 49(63) |
| MLL-PTD |  |
| Present | 4(5) |
| Absent | 74(95) |
| MLL-translocation |  |
| Present | 5(6) |
| MLL-MLLT4 | 2(3) |
| MLLT10-PICALM | 2(3) |
| MLL-ELL | 1(1) |
| Absent | 73(94) |
| MYH11-CBFB |  |
| Present | 5(6) |
| Absent | 73(94) |
| BCR-ABL1 |  |
| Present | 2(3) |
| Absent | 76(97) |
| NUP98-NSD1 |  |
| Present | 2(3) |
| Absent | 76(97) |

Abbreviations: WBC, white blood cell; BM, bone marrow; FAB, French-American-British; MRD, matched related donor; MUD, matched unrelated donor; CR, complete remission.
